# The role of coffee and potential mediators in subclinical atherosclerosis: insights from Mendelian randomization study

**DOI:** 10.3389/fnut.2024.1405353

**Published:** 2024-07-25

**Authors:** Qiwen Yang, Yue Yuan, Diyang Lyu, Rui Zhuang, Donghua Xue, Chaofeng Niu, Liyong Ma, Lijing Zhang

**Affiliations:** ^1^Graduate School, Beijing University of Chinese Medicine, Beijing, China; ^2^Department of Cardiology, Dongzhimen Hospital, Beijing University of Chinese Medicine, Beijing, China; ^3^Food Science Editorial Department, Beijing Academy of Food Science, Beijing, China

**Keywords:** coffee, coronary artery calcium, subclinical atherosclerosis, body mass index, Mendelian randomization

## Abstract

**Background and aims:**

Coffee contains many bioactive compounds, and its inconsistent association with subclinical atherosclerosis has been reported in observational studies. In this Mendelian randomization study, we investigated whether genetically predicted coffee consumption is associated with subclinical atherosclerosis, as well as the role of potential mediators.

**Methods:**

We first conducted a two-sample Mendelian randomization analysis to examine the causal effect of coffee and its subtypes on subclinical atherosclerosis inferred from coronary artery calcification (CAC). Next, the significant results were validated using another independent dataset. Two-step Mendelian randomization analyses were utilized to evaluate the causal pathway from coffee to subclinical atherosclerosis through potential mediators, including blood pressure, blood lipids, body mass index, and glycated hemoglobin. Mendelian randomization analyses were performed using the multiplicative random effects inverse-variance weighted method as the main approach, followed by a series of complementary methods and sensitivity analyses.

**Results:**

Coffee, filtered coffee, and instant coffee were associated with the risk of CAC (β = 0.79, 95% CI: 0.12 to 1.47, *p* = 0.022; β = 0.66, 95% CI: 0.17 to 1.15, *p* = 0.008; β = 0.66, 95% CI: 0.20 to 1.13, *p* = 0.005; respectively). While no significant causal relationship was found between decaffeinated coffee and CAC (β = −1.32, 95% CI: −2.67 to 0.04, *p* = 0.056). The association between coffee and CAC was validated in the replication analysis (β = 0.27, 95% CI: 0.07 to 0.48, *p* = 0.009). Body mass index mediated 39.98% of the effect of coffee on CAC (95% CI: 9.78 to 70.19%, *p* = 0.009), and 5.79% of the effect of instant coffee on CAC (95% CI: 0.54 to 11.04%, *p* = 0.030).

**Conclusion:**

Our study suggests that coffee other than decaffeinated coffee increases the risk of subclinical atherosclerosis inferred from CAC. Body mass index mediated 39.98 and 5.79% of the causal effects of coffee and instant coffee on CAC, respectively. Coffee should be consumed with caution, especially in individuals with established cardiovascular risk factors, and decaffeinated coffee appears to be a safer choice.

## 1 Introduction

Although significant improvements in the prevention and treatment of atherosclerotic cardiovascular disease (ASCVD) have been made in recent decades, ASCVD remains the leading cause of morbidity and mortality worldwide (1, 2). The long latency period between atherosclerosis and the onset of ASCVD allows the progression of atherosclerosis to be insidious for years (3). Therefore, the identification and management of atherosclerosis before it becomes symptomatic is an important public health goal with growing concerns. Asymptomatic atherosclerosis is widely known as subclinical atherosclerosis, which means the presence of atheromatous disease before there are any signs, symptoms, or events attributable to clinically manifest atherosclerotic disease (4). It is an early indicator of atherosclerotic burden, and it is important because timely intervention could prevent future cardiovascular morbidity and mortality (4). The use of non-invasive measurements such as arterial stiffness index (ASI), coronary artery calcification (CAC) scan and carotid intima-media thickness (cIMT) have allowed to infer the presence of subclinical atherosclerosis in the major conduit arteries (5–8).

One of the most important ways to prevent ASCVD is to promote a healthy lifestyle throughout life (2). As a part of the diet, coffee is one of the most popular beverages worldwide, with an average daily consumption of about four cups of coffee *per capita* in Europe (9). As a mixture of several biologically active compounds, including caffeine, diterpenes, chlorogenic acids, and melanoidins, coffee may bring both benefits and risks to the cardiovascular system (10, 11).

To date, cardiovascular health outcomes associated with coffee consumption have been widely studied, but some aspects remain controversial. The relationship between coffee consumption and the risk of subclinical atherosclerosis has been inconsistent in observational studies. These results suggest a positive (12–14), negative (15–17), and no correlation (18–20), respectively. Moreover, whether the association between coffee consumption and subclinical atherosclerosis is causal has not yet been established. Clarifying these issues could facilitate public health and clinical decision-making.

Mendelian randomization (MR) utilizes genetic variations as instrumental variables (IVs) to determine whether the association between exposure and outcome is consistent with a causal inference, which has the major advantage of being not susceptible to potential confounders and reverse causality (21). MR is useful in the above situations where randomized controlled trials (RCTs) are not feasible or practical (22). Another advantage of MR is the potential for larger sample sizes, which provides sufficient statistical power to detect causal relationships (23). CAC is a valid indicator of subclinical atherosclerosis, and it also provides independent predictive information on the risk of CVD (8, 24–26). Therefore, we conducted the first comprehensive MR analysis aiming to investigate the causal association between coffee consumption and subclinical atherosclerosis inferred from CAC.

## 2 Materials and methods

### 2.1 Study design

**Figure 1:**
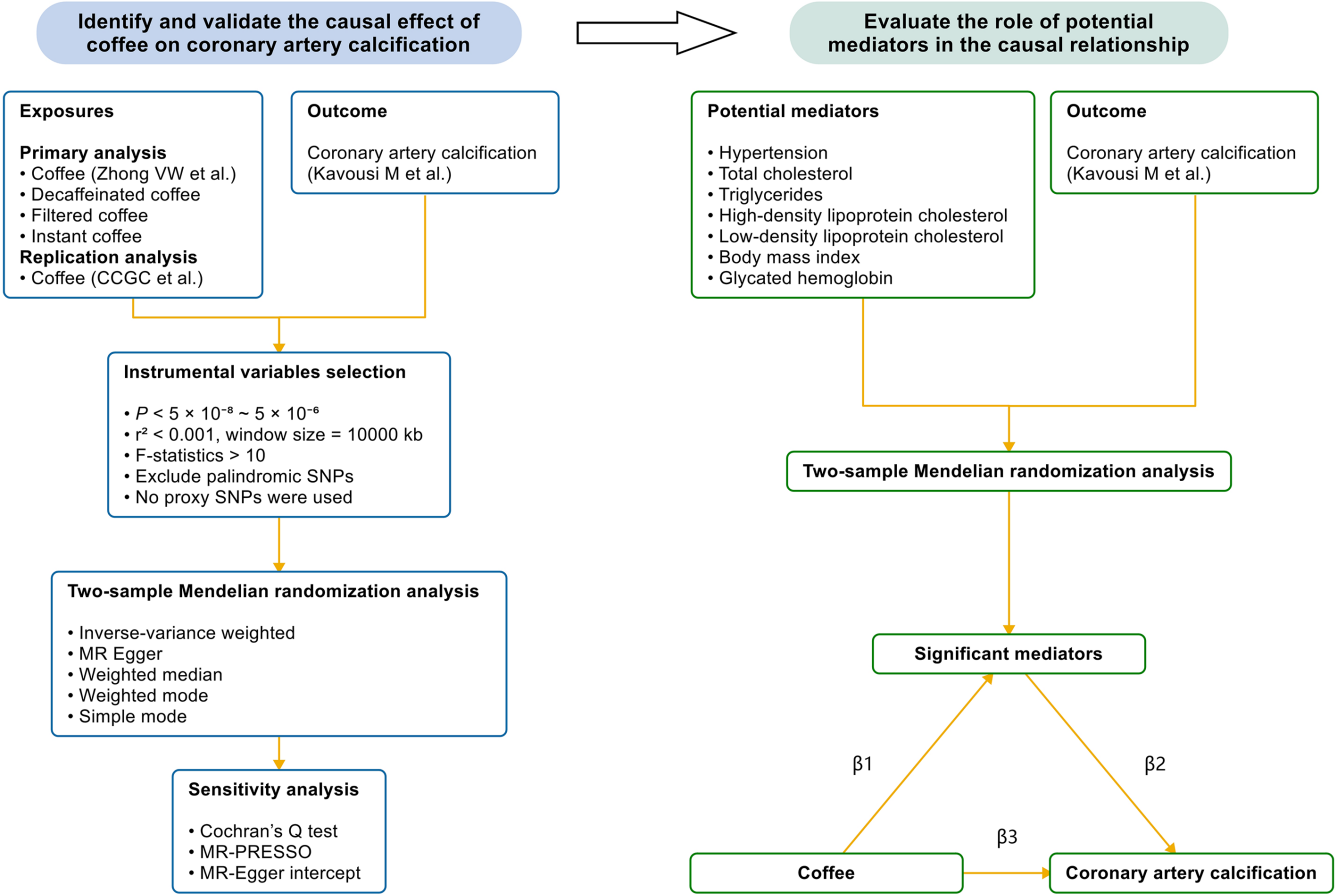
The study design. We first performed a two-sample Mendelian randomization analysis to identify the causal effect of coffee and its subtypes on CAC, which is a reliable indicator of subclinical atherosclerosis. Next, we validated the causal relationship by utilizing another GWAS of coffee. Finally, we performed a mediation analysis to evaluate the effect of potential mediators in the causal relationship between coffee and CAC. Coffee and its subtypes with significant causal effects on CAC were included in the two-step Mendelian randomization analysis. CCGC, the Coffee and Caffeine Genetics Consortium; SNP, single nucleotide polymorphism; MR-PRESSO, Mendelian randomization pleiotropy residual sum and outlier; β1, the significant effect of coffee on mediators; β2, the significant effect of mediators on CAC; β3, the significant total effect of coffee on CAC.

This MR study was based on publicly available genetic data obtained from the genome-wide association studies (GWAS) database and followed the STROBE-MR guidelines (27). Figure 1 demonstrates the study design. We first conducted a two-sample MR to examine the causal effect of coffee and its subtypes on subclinical atherosclerosis. Next, we validated the significant results in the replication analysis. Finally, we evaluated the causal pathway from coffee to subclinical atherosclerosis through potential mediators, using a two-step MR design. Blood pressure, blood lipids, body mass index and glycated hemoglobin, as common cardiovascular risk factors, were included in the two-step MR analysis.

CAC is one of the few reliable indicators of the presence of subclinical atherosclerosis (8), and therefore it was used to infer subclinical atherosclerosis. Single nucleotide polymorphisms (SNPs) associated with exposure were employed as IVs, which must meet three key assumptions: (1) IVs are associated with exposure, (2) IVs are not associated with confounders, and (3) IVs influence the outcome only through exposure (21).

The detailed summary information of GWAS data sources is presented in Supplementary Table S1. Data sources were carefully selected to minimize participant overlap, which could lead to inflated type I errors (Supplementary Table S2).

### 2.2 Exposure data source

Coffee consumption is a common modifiable exposure that may not be highly heritable, and identifying coffee consumption through self-reported measurements may introduce selection, recall, and measurement biases. Therefore, to mitigate the impact of these limitations, replication analysis across independent datasets is necessary (28). Furthermore, the effects of coffee may differ depending on the type (10, 29–31), hence analyses on coffee subtypes were also included.

In the primary analysis, summary data were extracted from a GWAS meta-analysis of beverage consumption conducted by Zhong et al., involving up to 375,833 participants of European ancestry (~89% from the UK Biobank) (32). Self-reported measurements of coffee have been detailed in the study (32). Age, sex, body mass index (BMI), and the top 20 principal components were adjusted (32).

Summary data for coffee subtypes including decaffeinated, filtered, and instant coffee were obtained from MRC-IEU UK Biobank OpenGWAS, which involved 64,949 European individuals (33). The coffee subtypes in the UK Biobank were determined by questionnaires on the most frequent coffee type consumed. Age, sex, and the first 10 principal components were adjusted.

Then, we validated the results of the primary analysis in another GWAS meta-analysis of coffee published by the Coffee and Caffeine Genetics Consortium (CCGC) (34). In the discovery stage (stage 1), a meta-analysis of GWAS summary data from 28 cohorts was performed to detect SNPs associated with coffee consumption (cups/day), incorporating 91,462 individuals of European ancestry. Self-reported measurements of coffee consumption were detailed in the meta-analysis (34). Age, smoking status, and, when applicable, sex, case–control status, study site, family structure, and/or study-specific principal components of the population substructure were adjusted (34). The estimated overlap of coffee from Coffee and Caffeine Genetics Consortium et al. with coffee from Zhong et al. was less than 6%.

### 2.3 Outcome data source

Genetic associations with CAC were obtained from the largest GWAS meta-analysis conducted by Kavousi et al., which evaluated CAC quantity expressed in Agatston scores (35) from 35,776 individuals of European and African ancestry across cohorts in the Cohorts for Heart and Aging Research in Genomic Epidemiology (CHARGE) consortium and collaborating cohorts (36). Only data from 26,909 European individuals were used in our MR study. CAC scores were evaluated using computed tomography (36). Age, sex, and the first 10 principal components were adjusted (36).

### 2.4 Mediator data source

Summary data for hypertension were obtained from the FinnGen study (102,864 cases and 289,117 controls of European ancestry) (37). The International Classification of Diseases diagnosis (ICD) codes (I10, I11, I12, I13, I14, I15, and I67.4) were used to define essential hypertension. Age, sex, 10 principal components, and genotyping batch were adjusted.

Summary data for blood lipids [total cholesterol (TC), triglycerides (TG), high-density lipoprotein cholesterol (HDL-C) and low-density lipoprotein cholesterol (LDL-C)] were obtained from a GWAS meta-analysis of cohorts in the Global Lipids Genetics Consortium (GLGC), which involved up to 188,577 European individuals (38). In most of the included studies, blood lipid levels were typically measured after >8 h of fasting and individuals known to be on lipid-lowering medication were excluded when possible (38). Age, age^2^ and sex were adjusted (38).

Summary data for body mass index (BMI) were obtained from a GWAS meta-analysis of cohorts in the Genetic Investigation of Anthropometric Traits (GIANT) consortium, including 339,224 individuals (~94.97% European ancestry) (39). BMI measured or self-reported weight in kg per height in meters squared was adjusted for age, age^2^, and any necessary study-specific covariates (39).

Summary data for glycated hemoglobin (HbA1C) were obtained from a transethnic GWAS meta-analysis of cohorts in the Meta-Analyses of Glucose and Insulin-related traits Consortium (MAGIC) (40). Only data from 123,665 European individuals were used in our MR study. All participants were free of diabetes and HbA1C was measured using fasting blood in most cohorts (40). Age, sex and study-specific covariates were adjusted (40).

### 2.5 Instrumental variable selection

The conventional threshold for the selection of significant genome-wide association SNPs is *p* < 5 × 10^−8^. However, this approach could be problematic when the number of selected SNPs is too small, which lead to underpowered analyses or, in some cases, inflated results (41). With this in mind, we set a relatively liberal *p* value threshold when necessary to ensure no less than 3 SNPs were eligible for IVs, so as to maximize power to detect significant associations. More specifically, the *p* value threshold was set at 5 × 10^−6^ for decaffeinated coffee, while 5 × 10^−7^ for filtered coffee, instant coffee and coffee (Coffee and Caffeine Genetics Consortium et al.). Then 5 × 10^−8^ was applied to coffee (Zhong et al.) and mediators. Second, to ensure independence between SNPs, the threshold of linkage disequilibrium was *r*^2^ < 0.001 with a 10,000 kb window. A European reference panel was used for clumping. Third, SNPs with F-statistics <10 were conventionally excluded to avoid weak instrumental bias, calculated by the formula *F* = (*N*−*K*−1) / *K* × *R*^2^ / (1−*R*^2^), where *R*^2^ is the proportion of variance in the phenotype explained by SNPs, K represents the number of SNPs, and N is the sample size (42). *R*^2^ was calculated by the formula *R*^2^ = 2 × β^2^ × EAF × (1−EAF), where EAF is the effect allele frequency and β is the estimated genetic effect (43). Lastly, we excluded palindromic SNPs that could not be certain about their forward strand in the process of harmonization in order to reduce the risk of errors (44). IVs absent in the outcome data sources were excluded from subsequent analyses, rather than using proxy SNPs.

### 2.6 Statistical analysis

The multiplicative random effects inverse-variance weighted (IVW) method was used as the primary approach to estimate the causal effect of coffee on CAC (45). Other methods, including the MR Egger, weighted median, weighted mode, and simple mode, provided complementary information. The IVW method assumes that all SNPs are valid and thus may produce the most precise estimate, and the results will be unbiased when horizontal pleiotropy is absent (46). The MR Egger method allows horizontal pleiotropy to be present in more than 50% of IVs (47). The weighted median method allows for a correct estimation of causal associations when up to 50% of IVs are invalid (48). A *p* value < 0.05 suggested a significant causal effect after applying the false discovery rate (FDR) correction using the Benjamini–Hochberg method.

Three sensitivity analyses were performed to establish the robustness of the MR results: Cochran’s Q test, the MR Egger intercept, and the MR pleiotropy residual sum and outlier (MR-PRESSO). Cochran’s Q test can detect heterogeneity using the IVW and MR Egger methods, and heterogeneity exists when *p* < 0.05 (49). Horizontal pleiotropy was evaluated by the MR Egger intercept, with *p* < 0.05 indicating its presence (47). MR-PRESSO served to identify outliers, and provided a causal estimate after removing outliers (50). The MR-PRESSO global test was employed in both primary and replication analyses as a complementary method for the detection of potential horizontal pleiotropy, with a threshold of *p* < 0.05.

To understand the potential causal mechanisms between coffee and CAC, a mediation analysis was performed. Coffee and its subtypes with significant causal effects on CAC were included in the two-step MR analysis. Hypertension, TC, TG, HDL-C, LDL-C, BMI and HbA1C were selected as potential mediators. We first explored the causal effects of potential mediators on CAC to identify significant mediators. Then, we analyzed the causal relationship between coffee and significant mediators (Figure 1). The proportion of the total effect mediated by mediators was estimated by dividing the indirect effect by the total effect (β1 × β2/β3). β1 represents the significant effect of coffee on mediators; β2 represents the significant effect of mediators on CAC; and β3 represents the significant total effect of coffee on CAC. Standard errors and 95% confidence interval were derived using the bootstrap method and effect estimates were obtained from two-sample MR analysis (51).

All analyses were performed using the “TwoSampleMR” (version 0.6.3) and “MR-PRESSO” (version 1.0) packages in the R software (version 4.3.3).

## 3 Results

The characteristics of IVs associated with coffee and CAC were summarized in Supplementary Table S3. The number of IVs ranged from 4 to 26, and all IVs showed F-statistics greater than 10.

**Figure 2:**
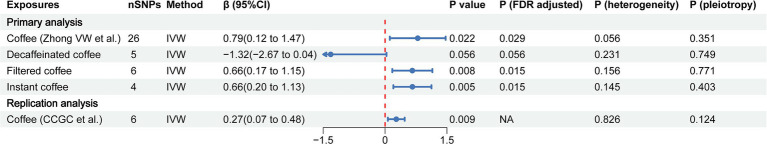
The forest plot of causal estimates between coffee and CAC in both primary and replication analyses. The heterogeneity test was performed using MR Egger method and pleiotropy was evaluated by MR Egger intercept. FDR, the false discovery rate correction; nSNPs, number of single nucleotide polymorphisms used for estimating the causal effects; CI, confidence interval; IVW, inverse-variance weighted; CCGC, the Coffee and Caffeine Genetics Consortium.

In the primary analysis, we identified that coffee, filtered coffee and instant coffee were associated with the risk of CAC based on the IVW method (β = 0.79, 95% CI: 0.12 to 1.47, *p* = 0.022; β = 0.66, 95% CI: 0.17 to 1.15, *p* = 0.008; β = 0.66, 95% CI: 0.20 to 1.13, *p* = 0.005; respectively) (Figure 2). The causal relationships remained significant after FDR correction. Other complementary MR methods including the MR Egger, weighted median, weighted mode, and simple mode confirmed the robustness of the IVW results (Table 1). However, no significant causal relationship was found between decaffeinated coffee and CAC using the IVW method (β = −1.32, 95% CI: −2.67 to 0.04, *p* = 0.056) or other complementary MR methods. We subsequently validated this association in the replication analysis using the IVW method (β = 0.27, 95% CI: 0.07 to 0.48, *p* = 0.009) and other complementary MR methods (Figure 2; Supplementary Table S4). The MR results in the primary and replication analyses were supported by sensitivity analyses and no evidence of heterogeneity or pleiotropy was detected. Therefore, as the primary method, the IVW method produced the most precise and unbiased causal estimate.

**Table 1:** Mendelian randomization analyses of coffee and its subtypes on CAC.

Exposures	Sample size	nSNPs	Methods	β (95% CI)	*p* value	P (FDR adjusted)	P (heterogeneity)	P (pleiotropy)
Coffee (Zhong VW et al.)	335,909	26	MR Egger	1.36 (0.01 to 2.72)	0.061		0.056	0.351
		26	Weighted median	1.26 (0.46 to 2.06)	0.002			
		26	IVW	0.79 (0.12 to 1.47)	0.022	0.029	0.054	
		26	Simple mode	−1.14 (−3.17 to 0.88)	0.278			
		26	Weighted mode	1.24 (0.45 to 2.04)	0.005			
		26	MR-PRESSO					0.069
Decaffeinated coffee	64,949	5	MR Egger	−0.92 (−3.61 to 1.76)	0.549		0.231	0.749
		5	Weighted median	−1.61 (−3.32 to 0.09)	0.063			
		5	IVW	−1.32 (−2.67 to 0.04)	0.056	0.056	0.346	
		5	Simple mode	0.32 (−2.40 to 3.05)	0.827			
		5	Weighted mode	−2.08 (−4.23 to 0.07)	0.131			
		5	MR-PRESSO					0.360
Filtered coffee	64,949	6	MR Egger	1.26 (−2.56 to 5.08)	0.553		0.156	0.771
		6	Weighted median	0.85 (0.29 to 1.40)	0.003			
		6	IVW	0.66 (0.17 to 1.15)	0.008	0.015	0.235	
		6	Simple mode	0.97 (0.09 to 1.86)	0.084			
		6	Weighted mode	0.96 (0.18 to 1.74)	0.060			
		6	MR-PRESSO					0.307
Instant coffee	64,949	4	MR Egger	2.93 (−1.31 to 7.17)	0.309		0.145	0.403
		4	Weighted median	0.67 (0.24 to 1.11)	0.002			
		4	IVW	0.66 (0.20 to 1.13)	0.005	0.015	0.111	
		4	Simple mode	0.75 (0.03 to 1.46)	0.135			
		4	Weighted mode	0.68 (0.07 to 1.29)	0.117			
		4	MR-PRESSO					0.230

Beta (β), 95% confidence interval (CI), and p values were calculated for the respective method of MR analysis. The heterogeneity was tested by Cochran’s Q, including the IVW and MR Egger methods. Horizontal pleiotropy was evaluated by MR Egger intercept and MR-PRESSO global test. nSNPs, number of single nucleotide polymorphisms; FDR, the false discovery rate correction; MR, Mendelian randomization; IVW, inverse-variance weighted; MR-PRESSO, MR pleiotropy residual sum and outlier.

**Figure 3:**
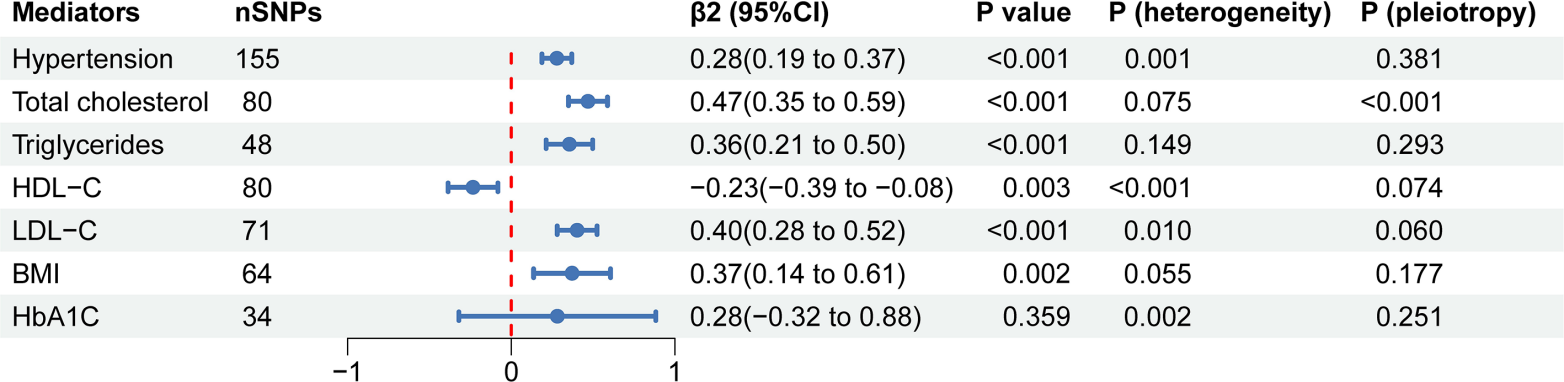
The forest plot of causal estimates between mediators and CAC using the IVW method. The heterogeneity test was performed using MR Egger method and pleiotropy was evaluated by MR Egger intercept. nSNPs, number of single nucleotide polymorphisms used for estimating the causal effects; β2, the effect of mediators on CAC; CI, confidence interval; HDL-C, high-density lipoprotein cholesterol; LDL-C, low-density lipoprotein cholesterol; BMI, body mass index; HbA1C, glycated hemoglobin.

**Figure 4:**
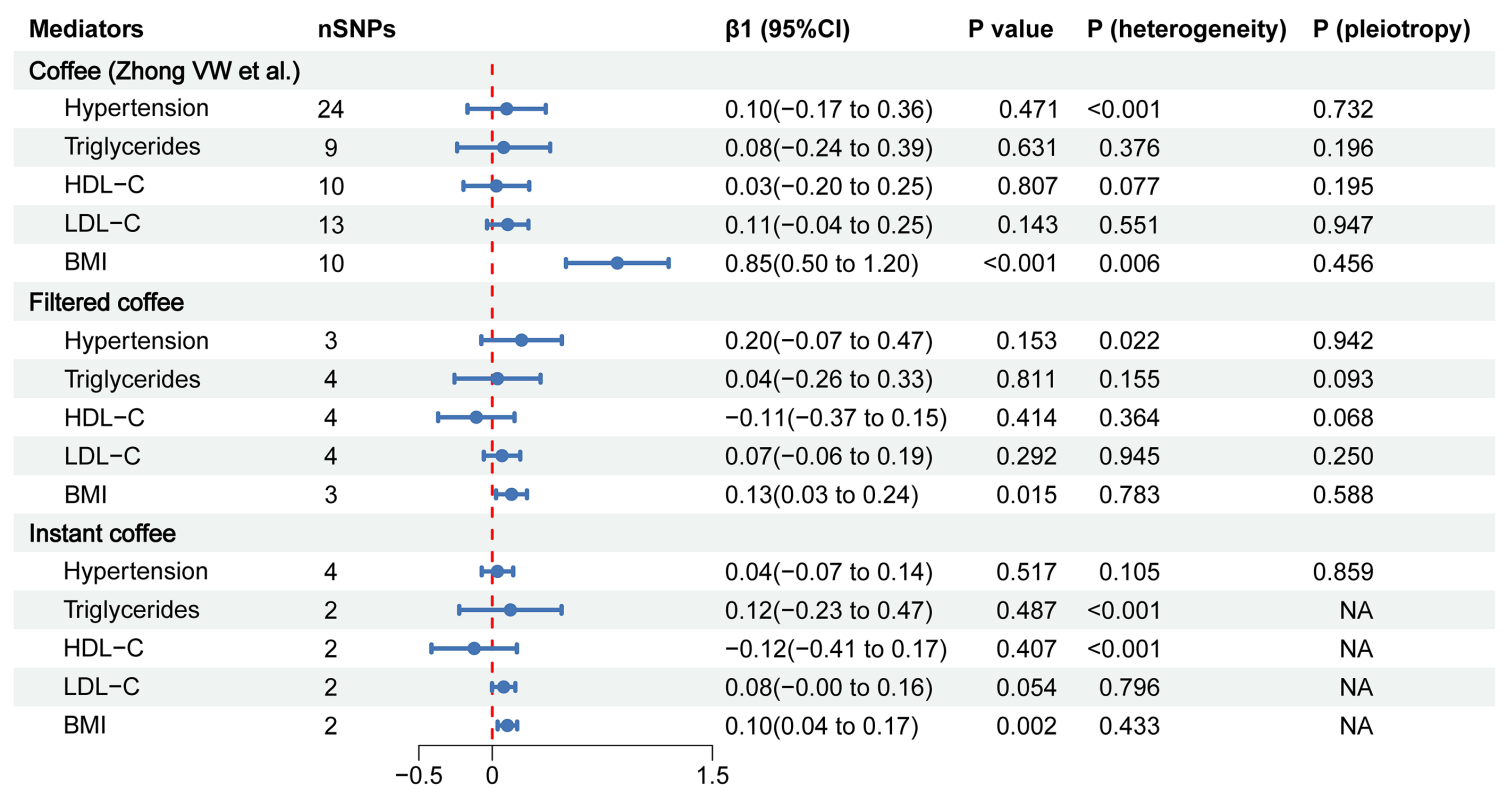
The forest plot of causal estimates between coffee and mediators using the IVW method. The heterogeneity test was performed using MR Egger method and pleiotropy was evaluated by MR Egger intercept. When only 2 instrumental variables existed, the pleiotropy test was not applicable, and the heterogeneity was evaluated by the IVW method. nSNPs, number of single nucleotide polymorphisms used for estimating the causal effects; β1, the effect of coffee on mediators; CI, confidence interval; HDL-C, high-density lipoprotein cholesterol; LDL-C, low-density lipoprotein cholesterol; BMI, body mass index.

**Figure 5:**
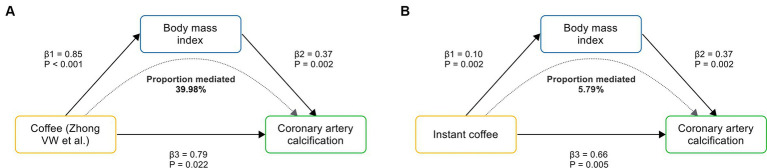
The causal pathway mediated by BMI. **(A)** BMI mediates the causal effect of coffee (Zhong VW et al.) on CAC. **(B)** BMI mediates the causal effect of instant coffee on CAC. β1 represents the causal effect of coffee on BMI; β2 represents the causal effect of BMI on CAC; and β3 represents the total causal effect of coffee on CAC.

To evaluate the role of potential mediators in the causal relationship between coffee and CAC, we conducted a two-step MR analysis. We first identified 6 significant mediators, including hypertension (β = 0.28, 95% CI: 0.19 to 0.37, *p* < 0.001), TC (β = 0.47, 95% CI: 0.35 to 0.59, *p* < 0.001), TG (β = 0.36, 95% CI: 0.21 to 0.50, *p* < 0.001), HDL-C (β = −0.23, 95% CI: −0.39 to −0.08, *p* = 0.003), LDL-C (β = 0.40, 95% CI: 0.28 to 0.52, *p* < 0.001) and BMI (β = 0.37, 95% CI: 0.14 to 0.61, *p* = 0.002) (Figure 3; Supplementary Table S5). While no significant association was found between HbA1C and CAC (β = 0.28, 95% CI: −0.32 to 0.88, *p* = 0.359). Cochran’s Q test indicated heterogeneity in the effects of hypertension, HDL-C, LDL-C and HbA1C on CAC. The MR Egger intercept test showed pleiotropy between TC and CAC (*p* < 0.001), which was inconsistent with the key assumptions of the MR study (21). Therefore, TC was excluded from subsequent MR analysis. Next, we tested the causal relationship between coffee and the remaining 5 significant mediators (Figure 4). Coffee (Zhong et al.), filtered coffee and instant coffee were all significantly associated with BMI using the IVW method (β = 0.85, 95% CI: 0.50 to 1.20, *p* < 0.001; β = 0.13, 95% CI: 0.03 to 0.24, *p* = 0.015; β = 0.10, 95% CI: 0.04 to 0.17, *p* = 0.002; respectively). In the subsequent sensitivity analyses, there was no evidence of pleiotropy, while heterogeneity was detected in the effects of coffee (Zhong et al.) on BMI (Supplementary Tables S6–S8). BMI mediated 39.98% of the effect of coffee (Zhong et al.) on CAC (95% CI: 9.78 to 70.19%, *p* = 0.009), and 5.79% of the effect of instant coffee on CAC (95% CI: 0.54 to 11.04%, *p* = 0.030) (Table 2). While the mediating effect of filtered coffee on CAC via BMI was non-significant (*p* = 0.055). The causal pathway mediated by BMI is visualized in Figure 5.

**Table 2:** Estimated proportion of the total effect mediated by mediator(s).

Mediating pathway	Indirect effect (95% CI)	Proportion mediated (95% CI)	*p* value
Coffee (Zhong et al.) to CAC via BMI	0.317 (0.078, 0.556)	39.98% (9.78, 70.19%)	0.009
Filtered coffee to CAC via BMI	0.049 (−0.001, 0.099)	7.39% (−0.22, 14.99%)	0.055
Instant coffee to CAC via BMI	0.038 (0.004, 0.073)	5.79% (0.54, 11.04%)	0.030

95% CI were derived using the bootstrap method.CI, confidence interval; CAC, coronary artery calcification; BMI, body mass index.

## 4 Discussion

This comprehensive MR study revealed the causal effects of coffee, filtered coffee and instant coffee on the risk of subclinical atherosclerosis inferred from CAC. The subsequent replication analysis reinforced the causal relationships. Conversely, no causal relationship was found between decaffeinated coffee and CAC. Next, we conducted a two-step MR analysis to investigate potential mediators along the pathway linking coffee to CAC. Notably, BMI mediated 39.98 and 5.79% of the causal effect of coffee and instant coffee on CAC, respectively.

### 4.1 Findings from previous studies

The role of coffee in CVD has been frequently discussed. However, coffee consumption and subclinical atherosclerosis have received less attention, and their relationship remains controversial. In a large cross-sectional study of 25,138 adults free of clinically evident CVD, coffee consumption was associated with a lower prevalence of CAC, and the association was U-shaped, with about 3–5 cups/day having the lowest prevalence (15). In another cross-sectional study, a significantly inverse association was observed between coffee consumption (>3 cups/d) and CAC scores (16). Interestingly, contradictory results even appeared within one study. In the Rotterdam study, severe CAC (CAC scores ≥100) in women was significantly reduced for more than 3 cups/day of coffee consumption compared with a daily consumption of 3 cups or less. But researchers found an unexplained increased risk of severe CAC among non-smoking men (52). The results also suggested that the interaction between smoking status and sex might play a role in the effect of coffee on CAC. Other findings from the CARDIA study (18) and the MESA study (19, 20) suggested no association between coffee consumption and CAC. The tangled relationship between coffee and subclinical atherosclerosis might have been caused by residual confounding and measurement errors such as unfavorable lifestyles, coffee-correlated traits (e.g., type of beans, preparation method, brew strength, additional sugar or milk, and definition of “a cup”), population stratification, and recall bias.

Previous studies have suggested that blood pressure (53), lipids (54–56), and glucose (57–59) are associated with the risk of CAC, which is broadly consistent with our MR results (Figure 3). Although we found that HbA1C was associated with CAC with the same effect trend as that in previous studies, the MR result was not statistically significant. This could be due to the fact that none of the participants in the GWAS data had diabetes (40), and the relationship between HbA1C and CAC might be non-linear (57). The relationship between BMI and CAC could be controversial (60–62), and our MR results confirmed the causal effect of BMI on CAC, which provided clues for the prevention and treatment of CAC.

MR analysis has been progressively employed in recent years to investigate the relationship between coffee and CVD, while not all MR studies have taken coffee subtypes into account. It is noteworthy that few MR studies have investigated the relationship between coffee and subclinical atherosclerosis. Unfortunately, previous MR studies did not provide solid evidence of causal relationships between coffee and CVD. Yuan et al. (63) found no causal effect of coffee on 15 CVD outcomes, including atrial fibrillation (AF), coronary artery disease (CAD), heart failure (HF) and stroke, using IVs from Zhong et al. (32) and outcome data from the UK Biobank and FinnGen study. Results were similar when using data from other GWAS (64–67). However, another MR analysis revealed different findings. To evaluate the causal effect of coffee on CAD, Zhang et al. (68) used IVs from Zhong et al. (32) and found that coffee was associated with an increased risk of CAD using outcome data from the CARDIoGRAMplusC4D consortium, whereas the association was non-significant using outcome data from the FinnGen study. Considering that Yuan et al. and Zhang et al. used consistent criteria for selecting IVs and rigorous methodology, the different results could be attributed to the selection of outcome data. Thus, the relationship between coffee and CVD needs to be further validated in larger GWAS or in non-European populations. In contrast, the results of observational studies suggested that coffee reduces the risk of CVD (69). Such discrepancy is partly attributed to the inevitable confounders. Furthermore, many previous non-significant MR results may indicate the lack of a direct association between coffee and CVD. Our robust findings highlighted the causal effect of coffee on CAC, helping clarify the underlying association between coffee and CVD.

### 4.2 Potential mechanisms

Several potential mechanisms support the positive association between coffee consumption and the risk of subclinical atherosclerosis. First is the effect on blood pressure. Caffeine is the most well-known compound in coffee, and a meta-analysis has shown that caffeine (pure caffeine, not in the form of coffee) leads to higher blood pressure (70). Caffeine is primarily metabolized by cytochrome P450 enzyme 1A2 (CYP1A2), the activity of which is partly inherited (71), so people with slower metabolism are at increased risk of hypertension (72). Second, caffeine has a detrimental effect on arterial stiffness, which could be attributed to the increase in sympathetic activity, the release of catecholamine, the antagonism of endogenous adenosine, and the stimulation of the upstream central nervous system (11, 73). Third is the effect on blood lipids. Kahweol and cafestol contribute to the bitter taste of coffee, and their content in coffee depends on the brewing technique. They are abundant in boiled and unfiltered coffee, but negligible in filtered and instant coffee (10). Evidence from meta-analysis indicates that consumption of unfiltered, but not filtered, coffee increases serum levels of TC and LDL-C (29, 30). All these factors may play a role in the pathogenesis of atherosclerosis. It also cannot be ignored that coffee is a complex mixture with diverse effects. For example, consuming caffeinated coffee (rather than pure caffeine) does not have a significant effect on blood pressure (31), probably because other components of coffee (e.g., chlorogenic acid) counteract the blood pressure-raising effect of caffeine (74). In addition, kahweol and cafestol can also enhance endogenous defense systems against oxidative damage, which contributes to the pathogenesis of atherosclerosis (75, 76).

In our primary analysis, decaffeinated coffee was found to have no causal effect on CAC compared with other types of coffee, suggesting that caffeine is probably a dominant factor that increases the risk of CAC (Figure 2). Blood pressure (77, 78) and lipids (29, 30) are one of the major controversies over the effects of coffee on cardiovascular health. Our MR results suggest that the combined effects of bioactive substances in coffee on blood pressure and lipids may be neutral (Figure 4). In addition, chlorogenic acid and caffeine have been found to be beneficial for reducing BMI (79, 80). While the common inclusion of artificial sweetener and sugar in coffee (especially in instant coffee) is associated with higher BMI (81, 82). There are mixed results from previous studies of the effects of coffee on BMI (81, 83, 84). Our findings support that coffee, filtered coffee and instant coffee can lead to increased BMI, which is consistent with the results of another MR study using different GWAS data sources (Figure 4) (85). However, the mediating effect of BMI between filtered coffee and CAC is non-significant (Table 2). The potential pathway of filtered coffee to increased CAC risk requires further investigation.

### 4.3 Clinical implications

Our MR study suggests that coffee leads to an increased risk of subclinical atherosclerosis inferred from CAC, and BMI mediates up to 39.98% of the effect of coffee on CAC. While decaffeinated coffee has no such adverse effect. These causal relationships can help to elucidate the role of coffee in the prevention of atherosclerosis. We recommend that coffee should be consumed with caution, especially in individuals with established cardiovascular risk factors, including subclinical atherosclerosis and obesity. Decaffeinated coffee without artificial sweeteners or sugar appears to be a safer substitute for other types of coffee.

The active compounds in coffee have both beneficial and adverse effects on the cardiovascular system. The concentrations of these compounds are associated with coffee-correlated traits, such as the type of beans, preparation method, and brew strength, which ultimately shape the combined effects of coffee. Further clinical or MR studies need to take more coffee-correlated traits into methodologic considerations, which will facilitate a deeper understanding of the effects of coffee on subclinical atherosclerosis and CVD.

### 4.4 Strengths and limitations

Our study has several strengths. The major merit is the MR design, which can make reasonable inferences about potential causality and avoid reverse causation or confounding biases. To ensure the robustness of the results, we carefully chose data sources and kept participant overlap to a small percentage to avoid inflated Type I error rates (86) (Supplementary Table S2). We analyzed the causal effect of different types of coffee on CAC and performed a replication analysis to enhance the reliability of the results. Next, we conducted a two-step MR analysis to further quantify the proportion of the exposure’s effect attributable to potential mediators, which improved the clinical implications of our findings. Sensitivity analyses were used to validate the robustness of our results.

Several limitations are inevitable. First, heterogeneity was detected in two-step MR analyses. For this we used multiplicative random effects IVW method as the primary method. This is not only because it is the most efficient analysis method with valid IVs but also because it provides consistent evidence supporting a causal effect, even accounting for heterogeneity in the causal estimates (45). This points to the limited impact of heterogeneity on our findings. Second, coffee consumption is self-reported at baseline and usually changes over a lifetime, whereas genetic variation is fixed at conception. Therefore, we were unable to assess the effect of coffee consumption on the risk of subclinical atherosclerosis at different stages of life. Third, we included data sources of European ancestry to minimize population stratification. This also restricted us from extrapolating our findings to non-European populations. Lastly, we were unable to assess possible J-shaped or U-shaped associations between coffee consumption and the risk of subclinical atherosclerosis, as observed in previous studies (13, 15).

## 5 Conclusion

Our MR study supports that coffee other than decaffeinated coffee leads to an increased risk of subclinical atherosclerosis inferred from CAC. BMI mediated 39.98 and 5.79% of the causal effects of coffee and instant coffee on CAC, respectively. We recommend that coffee should be consumed with caution, especially in individuals with established cardiovascular risk factors, including subclinical atherosclerosis and obesity. Decaffeinated coffee without artificial sweeteners or sugar appears to be a safer substitute for other types of coffee, since it has no such adverse effect. Further clinical or MR studies need to take more coffee-correlated traits into methodologic considerations, which will facilitate a deeper understanding of the effects of coffee on subclinical atherosclerosis and CVD.

## Data availability statement

The original contributions presented in the study are included in the article/Supplementary material, further inquiries can be directed to the corresponding author.

## Ethics statement

In this Mendelian randomization study, all data were extracted from publicly available GWAS databases that had previously obtained ethical approval and informed consent. Therefore, no further ethical approval was required. The studies were conducted in accordance with the local legislation and institutional requirements.

## Author contributions

QY: Conceptualization, Formal analysis, Methodology, Writing – original draft. YY: Conceptualization, Data curation, Writing – original draft. DL: Methodology, Writing – review & editing. RZ: Formal analysis, Writing – review & editing. DX: Visualization, Writing – review & editing. CN: Software, Writing – review & editing. LM: Funding acquisition, Writing – review & editing. LZ: Supervision, Writing – review & editing.
