# Supplementary material for: The role of coffee and potential mediators in subclinical atherosclerosis: insights from Mendelian randomization study
Source: Front Nutr. 2024 Jul 25;11:1405353. doi: 10.3389/fnut.2024.1405353 (PMC11309031; doi:10.3389/fnut.2024.1405353)
Supplement: Supplementary file 1 [file Data_Sheet_1.zip › Supplementary_Material.docx]

Supplementary Material

# Supplementary Tables

**Supplementary Table S1** Overview of the Data Sources Used in the Mendelian Randomization Analysis

**Supplementary Table S2** Estimated Overlap Between Data Sources

**Supplementary Table S3** Instrumental Variables of Coffee

**Supplementary Table S4** Mendelian Randomization and Sensitivity Analyses of Coffee on CAC in Replication Analysis

**Supplementary Table S5** Mendelian Randomization and Sensitivity Analyses of Mediators on CAC

**Supplementary Table S6** Mendelian Randomization and Sensitivity Analyses of Coffee (Zhong VW et al.) on Mediators

**Supplementary Table S7** Mendelian Randomization and Sensitivity Analyses of Filtered Coffee on Mediators

**Supplementary Table S8** Mendelian Randomization and Sensitivity Analyses of Instant Coffee on Mediators

**Supplementary Table S1** Overview of the Data Sources Used in the Mendelian Randomization Analysis

| **Phenotype** | **Year** | **GWAS ID** | **PMID** | **Ancestry** | **Sample size (cases/controls)** | **Unit** | **Consortium or study** |
| --- | --- | --- | --- | --- | --- | --- | --- |
| **Exposures** |  |  |  |  |  |  |  |
| Coffee consumption | 2019 | GCST008526 | 31046077 | European | 375833 | 50% change | ~89% from the UK Biobank |
| Decaffeinated coffee | 2018 | ukb-b-12558 | NA | European | 64949 | SD | MRC-IEU |
| Filtered coffee | 2018 | ukb-b-748 | NA | European | 64949 | SD | MRC-IEU |
| Instant coffee | 2018 | ukb-b-930 | NA | European | 64949 | SD | MRC-IEU |
| Coffee consumption (replication analysis) | 2015 | GCST002650 | 25288136 | European | 91462 | cups per day | CCGC |
| **Outcome** |  |  |  |  |  |  |  |
| Coronary artery calcification | 2023 | GCST90278456 | 37770635 | European | 26909 | SD | CHARGE consortium and collaborating cohorts |
| **Potential mediators** |  |  |  |  |  |  |  |
| Hypertension | 2023 | I9_HYPTENS | NA | European | 391981 (102864/289117) | One-unit in log-transformed odds ratio of hypertension | the FinnGen study |
| Total cholesterol | 2013 | ieu-a-301 | 24097068 | European | 187365 | SD | GLGC |
| Triglycerides | 2013 | ieu-a-302 | 24097068 | European | 177861 | SD | GLGC |
| High-density lipoprotein cholesterol | 2013 | ieu-a-299 | 24097068 | European | 187167 | SD | GLGC |
| Low-density lipoprotein cholesterol | 2013 | ieu-a-300 | 24097068 | European | 173082 | SD | GLGC |
| Body mass index | 2015 | ieu-a-2 | 25673413 | ~94.97% European | 339224 | SD | GIANT consortium |
| Glycated hemoglobin | 2017 | GCST007954 | 28898252 | European | 123665 | SD | MAGIC |

**Abbreviations:** SD, standard deviation; MRC-IEU, the MRC Integrative Epidemiology Unit; CCGC, the Coffee and Caffeine Genetics Consortium; CHARGE, the Cohorts for Heart and Aging Research in Genomic Epidemiology; GLGC, the Global Lipids Genetics Consortium; GIANT, the Genetic Investigation of Anthropometric Traits; MAGIC, the Meta-Analyses of Glucose and Insulin-related traits Consortium.

**Supplementary Table S2** Estimated Overlap Between Data Sources

| **Comparison between data sources** | | **Cohorts with overlap** | **Estimated Overlap proportion** |
| --- | --- | --- | --- |
| Coffee (Zhong VW et al.) | Coffee (CCGC et al.) | WGHS | <6% |
|  | Hypertension |  | 0 |
|  | Blood lipids |  | 0 |
|  | BMI | NHS, WGHS | <7% |
|  | HbA1C |  | 0 |
|  | CAC |  | 0 |
| Coffee subtypes | Coffee (CCGC et al.) |  | 0 |
|  | CAC |  | 0 |
| Coffee (CCGC et al.) | CAC | FamHS, FHS, MESA, RS-I, RS-II | <8% |
| CAC | Hypertension |  | 0 |
|  | Blood lipids |  | 0 |
|  | BMI | AGES, FamHS, LLS, NELSON, RS-I, YFS | <5% |
|  | HbA1C | FHS | <2% |

Blood lipids include data sources of total cholesterol, triglycerides, high-density lipoprotein cholesterol and low-density lipoprotein cholesterol. Coffee subtypes include data sources of decaffeinated coffee, filtered coffee and instant coffee. **Abbreviations:** BMI, body mass index; CAC, coronary artery calcification; HbA1C, glycated hemoglobin; CCGC, the Coffee and Caffeine Genetics Consortium.

**Supplementary Table S3** Instrumental Variables of Coffee

| **SNP** | **Effect allele** | **Other allele** | **Effect allele frequency** | **Beta** | **Standard error** | **P value** | **Sample size** | **R^2^** | **F-statistics** |
| --- | --- | --- | --- | --- | --- | --- | --- | --- | --- |
| **Coffee (Zhong VW et al.)** |  |  |  |  |  |  |  |  |  |
| rs10127720 | T | C | 0.260 | -0.011 | 0.002 | 1.39E-10 | 335909 | 5.02E-05 | 16.873 |
| rs1057868 | C | T | 0.715 | -0.019 | 0.002 | 2.44E-27 | 335909 | 1.42E-04 | 47.638 |
| rs10865548 | A | G | 0.172 | -0.015 | 0.002 | 2.35E-13 | 335909 | 6.50E-05 | 21.837 |
| rs10997940 | C | T | 0.596 | 0.009 | 0.002 | 3.09E-08 | 335909 | 3.73E-05 | 12.519 |
| rs117810762 | G | A | 0.982 | -0.035 | 0.006 | 4.66E-09 | 335909 | 4.28E-05 | 14.375 |
| rs117968677 | G | A | 0.975 | 0.029 | 0.005 | 3.67E-08 | 335909 | 4.06E-05 | 13.627 |
| rs1260326 | T | C | 0.393 | -0.013 | 0.002 | 2.92E-16 | 335909 | 8.09E-05 | 27.160 |
| rs16903275 | C | A | 0.844 | -0.015 | 0.002 | 2.46E-12 | 335909 | 5.94E-05 | 19.969 |
| rs17687539 | A | G | 0.791 | 0.010 | 0.002 | 4.69E-08 | 335909 | 3.63E-05 | 12.180 |
| rs2231142 | G | T | 0.886 | 0.015 | 0.002 | 1.42E-09 | 335909 | 4.43E-05 | 14.875 |
| rs2330783 | G | T | 0.986 | 0.043 | 0.007 | 6.20E-11 | 335909 | 5.17E-05 | 17.372 |
| rs2465037 | C | A | 0.657 | 0.011 | 0.002 | 5.56E-11 | 335909 | 5.25E-05 | 17.634 |
| rs2472297 | C | T | 0.733 | -0.044 | 0.002 | 8.28E-141 | 335909 | 7.71E-04 | 259.040 |
| rs2521501 | A | T | 0.677 | 0.010 | 0.002 | 1.44E-08 | 335909 | 3.95E-05 | 13.283 |
| rs2613458 | A | C | 0.279 | -0.010 | 0.002 | 4.60E-09 | 335909 | 4.23E-05 | 14.199 |
| rs34060476 | A | G | 0.866 | -0.018 | 0.002 | 8.05E-15 | 335909 | 7.32E-05 | 24.592 |
| rs4410790 | T | C | 0.366 | -0.038 | 0.002 | 4.73E-121 | 335909 | 6.64E-04 | 223.035 |
| rs56113850 | T | C | 0.423 | -0.012 | 0.002 | 2.47E-14 | 335909 | 7.03E-05 | 23.627 |
| rs57918684 | G | A | 0.845 | -0.013 | 0.002 | 6.57E-09 | 335909 | 4.10E-05 | 13.759 |
| rs597045 | A | T | 0.695 | 0.010 | 0.002 | 2.86E-09 | 335909 | 4.38E-05 | 14.708 |
| rs6062682 | C | T | 0.535 | -0.010 | 0.002 | 1.16E-10 | 335909 | 5.16E-05 | 17.347 |
| rs66723169 | C | A | 0.769 | -0.014 | 0.002 | 3.32E-14 | 335909 | 7.02E-05 | 23.584 |
| rs73075167 | A | T | 0.870 | 0.016 | 0.002 | 2.41E-11 | 335909 | 5.58E-05 | 18.755 |
| rs75347775 | G | A | 0.755 | -0.010 | 0.002 | 1.31E-08 | 335909 | 3.92E-05 | 13.162 |
| rs9937053 | G | A | 0.577 | -0.013 | 0.002 | 1.22E-16 | 335909 | 8.34E-05 | 28.012 |
| rs993885 | G | A | 0.633 | 0.010 | 0.002 | 1.01E-09 | 335909 | 4.55E-05 | 15.299 |
| **Decaffeinated coffee** |  |  |  |  |  |  |  |  |  |
| rs114776712 | T | A | 0.013 | 0.098 | 0.017 | 1.50E-08 | 64949 | 2.37E-04 | 15.390 |
| rs117663632 | T | C | 0.016 | 0.071 | 0.015 | 3.30E-06 | 64949 | 1.60E-04 | 10.375 |
| rs144796532 | T | C | 0.020 | 0.063 | 0.013 | 2.50E-06 | 64949 | 1.55E-04 | 10.075 |
| rs4953028 | A | G | 0.447 | 0.018 | 0.004 | 2.30E-06 | 64949 | 1.55E-04 | 10.081 |
| rs76521796 | T | C | 0.025 | 0.059 | 0.012 | 4.30E-07 | 64949 | 1.69E-04 | 10.980 |
| **Filtered coffee** |  |  |  |  |  |  |  |  |  |
| rs10468280 | G | A | 0.394 | 0.034 | 0.007 | 4.40E-07 | 64949 | 5.67E-04 | 36.847 |
| rs12535729 | T | C | 0.421 | 0.036 | 0.007 | 1.60E-07 | 64949 | 6.22E-04 | 40.410 |
| rs2470893 | T | C | 0.325 | 0.049 | 0.007 | 4.60E-12 | 64949 | 1.06E-03 | 68.986 |
| rs2830854 | G | A | 0.608 | 0.037 | 0.007 | 9.50E-08 | 64949 | 6.36E-04 | 41.336 |
| rs3004179 | G | A | 0.599 | -0.040 | 0.007 | 5.60E-09 | 64949 | 7.54E-04 | 48.999 |
| rs6968865 | T | A | 0.627 | 0.048 | 0.007 | 2.90E-12 | 64949 | 1.07E-03 | 69.310 |
| **Instant coffee** |  |  |  |  |  |  |  |  |  |
| rs1537362 | G | A | 0.158 | -0.078 | 0.015 | 2.40E-07 | 64949 | 1.61E-03 | 104.742 |
| rs2472297 | T | C | 0.258 | 0.073 | 0.012 | 4.60E-09 | 64949 | 2.05E-03 | 133.118 |
| rs2726351 | A | G | 0.098 | 0.099 | 0.019 | 3.00E-07 | 64949 | 1.72E-03 | 111.600 |
| rs6968554 | G | A | 0.633 | 0.074 | 0.011 | 9.40E-11 | 64949 | 2.52E-03 | 163.896 |
| **Coffee (CCGC et al.)** |  |  |  |  |  |  |  |  |  |
| rs1260326 | T | C | 0.410 | -0.040 | 0.010 | 1.06E-07 | 91407 | 7.74E-04 | 70.810 |
| rs17685 | A | G | 0.290 | 0.070 | 0.010 | 9.06E-14 | 85140 | 2.02E-03 | 172.141 |
| rs2049045 | C | G | 0.190 | -0.050 | 0.010 | 2.08E-07 | 89280 | 7.70E-04 | 68.752 |
| rs2472297 | T | C | 0.240 | 0.150 | 0.010 | 6.45E-47 | 87622 | 8.21E-03 | 725.137 |
| rs4410790 | T | C | 0.370 | -0.140 | 0.010 | 1.48E-57 | 86338 | 9.14E-03 | 796.172 |
| rs7800944 | T | C | 0.720 | -0.050 | 0.010 | 7.82E-09 | 87998 | 1.01E-03 | 88.789 |

**Abbreviations:** SNP, single nucleotide polymorphism; CCGC, the Coffee and Caffeine Genetics Consortium.

**Supplementary Table S4** Mendelian Randomization and Sensitivity Analyses of Coffee on CAC in Replication Analysis

| **Exposure** | **nSNPs** | **Methods** | **β (95% CI)** | **P value** | **P (heterogeneity)** | **P (pleiotropy)** |
| --- | --- | --- | --- | --- | --- | --- |
| Coffee (CCGC et al.) | 6 | MR Egger | 0.63 (0.22 to 1.05) | 4.04E-02 | 0.826 | 0.124 |
|  | 6 | Weighted median | 0.36 (0.12 to 0.59) | 2.66E-03 |  |  |
|  | 6 | IVW | 0.27 (0.07 to 0.48) | 8.92E-03 | 0.383 |  |
|  | 6 | Simple mode | 0.28 (-0.05 to 0.62) | 1.61E-01 |  |  |
|  | 6 | Weighted mode | 0.35 (0.11 to 0.58) | 3.27E-02 |  |  |
|  | 6 | MR-PRESSO |  |  |  | 0.481 |

Beta (β), 95% confidence interval (CI), and P values were calculated for the respective method of MR analysis. The heterogeneity was tested by Cochran’s Q, including IVW and MR Egger methods. Horizontal pleiotropy was evaluated by MR Egger intercept and MR-PRESSO global test. **Abbreviations:** nSNPs, number of single nucleotide polymorphisms; MR, Mendelian randomization; IVW, inverse-variance weighted; MR-PRESSO, MR pleiotropy residual sum and outlier.

**Supplementary Table S5** Mendelian Randomization and Sensitivity Analyses of Mediators on CAC

| **Exposures** | **nSNPs** | **Methods** | **β (95% CI)** | **P value** | **P (heterogeneity)** | **P (pleiotropy)** |
| --- | --- | --- | --- | --- | --- | --- |
| Hypertension | 155 | MR Egger | 0.16(-0.12 to 0.44) | 2.71E-01 | 0.001 | 0.381 |
|  | 155 | Weighted median | 0.24(0.12 to 0.36) | 6.57E-05 |  |  |
|  | 155 | IVW | 0.28(0.19 to 0.37) | 2.03E-09 | 0.001 |  |
|  | 155 | Simple mode | 0.33(0.02 to 0.63) | 3.65E-02 |  |  |
|  | 155 | Weighted mode | 0.24(0.00 to 0.48) | 4.77E-02 |  |  |
| Total cholesterol | 80 | MR Egger | 0.76(0.57 to 0.95) | 1.62E-11 | 0.075 | <0.001 |
|  | 80 | Weighted median | 0.55(0.39 to 0.71) | 6.10E-12 |  |  |
|  | 80 | IVW | 0.47(0.35 to 0.59) | 1.30E-14 | 0.006 |  |
|  | 80 | Simple mode | 0.42(0.10 to 0.74) | 1.27E-02 |  |  |
|  | 80 | Weighted mode | 0.60(0.45 to 0.75) | 1.42E-11 |  |  |
| Triglycerides | 48 | MR Egger | 0.26(0.05 to 0.48) | 2.19E-02 | 0.149 | 0.293 |
|  | 48 | Weighted median | 0.28(0.09 to 0.46) | 4.20E-03 |  |  |
|  | 48 | IVW | 0.36(0.21 to 0.50) | 9.87E-07 | 0.143 |  |
|  | 48 | Simple mode | 0.57(0.20 to 0.93) | 4.31E-03 |  |  |
|  | 48 | Weighted mode | 0.28(0.10 to 0.47) | 4.83E-03 |  |  |
| High-density lipoprotein cholesterol | 80 | MR Egger | -0.01(-0.30 to 0.27) | 9.23E-01 | <0.001 | 0.074 |
|  | 80 | Weighted median | -0.24(-0.44 to -0.04) | 1.82E-02 |  |  |
|  | 80 | IVW | -0.23(-0.39 to -0.08) | 2.61E-03 | <0.001 |  |
|  | 80 | Simple mode | -0.51(-0.97 to -0.06) | 2.81E-02 |  |  |
|  | 80 | Weighted mode | -0.20(-0.40 to 0.01) | 6.19E-02 |  |  |
| Low-density lipoprotein cholesterol | 71 | MR Egger | 0.54(0.36 to 0.73) | 2.46E-07 | 0.010 | 0.060 |
|  | 71 | Weighted median | 0.43(0.26 to 0.59) | 3.14E-07 |  |  |
|  | 71 | IVW | 0.40(0.28 to 0.52) | 9.65E-11 | 0.005 |  |
|  | 71 | Simple mode | 0.51(0.20 to 0.81) | 1.92E-03 |  |  |
|  | 71 | Weighted mode | 0.40(0.25 to 0.56) | 2.75E-06 |  |  |
| Body mass index | 64 | MR Egger | 0.81(0.14 to 1.48) | 2.12E-02 | 0.055 | 0.177 |
|  | 64 | Weighted median | 0.56(0.23 to 0.89) | 9.81E-04 |  |  |
|  | 64 | IVW | 0.37(0.14 to 0.61) | 1.95E-03 | 0.045 |  |
|  | 64 | Simple mode | 0.17(-0.62 to 0.97) | 6.69E-01 |  |  |
|  | 64 | Weighted mode | 0.72(0.19 to 1.26) | 1.02E-02 |  |  |
| Glycated hemoglobin | 34 | MR Egger | -0.32(-1.48 to 0.85) | 6.00E-01 | 0.002 | 0.251 |
|  | 34 | Weighted median | -0.23(-0.93 to 0.48) | 5.25E-01 |  |  |
|  | 34 | IVW | 0.28(-0.32 to 0.88) | 3.59E-01 | 0.001 |  |
|  | 34 | Simple mode | -0.02(-1.42 to 1.38) | 9.80E-01 |  |  |
|  | 34 | Weighted mode | -0.24(-1.05 to 0.57) | 5.68E-01 |  |  |

Beta (β), 95% confidence interval (CI), and P values were calculated for the respective method of MR analysis. The heterogeneity was tested by Cochran’s Q, including IVW and MR Egger methods. Horizontal pleiotropy was evaluated by MR Egger intercept. **Abbreviations:** nSNPs, number of single nucleotide polymorphisms; MR, Mendelian randomization; IVW, inverse-variance weighted.

**Supplementary Table S6** Mendelian Randomization and Sensitivity Analyses of Coffee (Zhong VW et al.) on Mediators

| **Exposures** | **nSNPs** | **Methods** | **β (95% CI)** | **P value** | **P (heterogeneity)** | **P (pleiotropy)** |
| --- | --- | --- | --- | --- | --- | --- |
| Hypertension | 24 | MR Egger | 0.02(-0.49 to 0.53) | 9.33E-01 | <0.001 | 0.732 |
|  | 24 | Weighted median | 0.03(-0.25 to 0.30) | 8.58E-01 |  |  |
|  | 24 | IVW | 0.10(-0.17 to 0.36) | 4.71E-01 | <0.001 |  |
|  | 24 | Simple mode | 0.04(-0.56 to 0.64) | 8.96E-01 |  |  |
|  | 24 | Weighted mode | 0.04(-0.17 to 0.25) | 7.09E-01 |  |  |
| Triglycerides | 9 | MR Egger | -0.61(-1.59 to 0.38) | 2.66E-01 | 0.376 | 0.196 |
|  | 9 | Weighted median | 0.02(-0.38 to 0.42) | 9.09E-01 |  |  |
|  | 9 | IVW | 0.08(-0.24 to 0.39) | 6.31E-01 | 0.285 |  |
|  | 9 | Simple mode | 0.01(-0.63 to 0.65) | 9.79E-01 |  |  |
|  | 9 | Weighted mode | -0.00(-0.68 to 0.67) | 9.97E-01 |  |  |
| High-density lipoprotein cholesterol | 10 | MR Egger | 0.24(-0.12 to 0.60) | 2.32E-01 | 0.077 | 0.195 |
|  | 10 | Weighted median | 0.08(-0.10 to 0.26) | 3.93E-01 |  |  |
|  | 10 | IVW | 0.03(-0.20 to 0.25) | 8.07E-01 | 0.038 |  |
|  | 10 | Simple mode | -0.10(-0.61 to 0.42) | 7.17E-01 |  |  |
|  | 10 | Weighted mode | 0.08(-0.10 to 0.26) | 4.20E-01 |  |  |
| Low-density lipoprotein cholesterol | 13 | MR Egger | 0.10(-0.16 to 0.35) | 4.65E-01 | 0.551 | 0.947 |
|  | 13 | Weighted median | 0.13(-0.05 to 0.30) | 1.55E-01 |  |  |
|  | 13 | IVW | 0.11(-0.04 to 0.25) | 1.43E-01 | 0.635 |  |
|  | 13 | Simple mode | 0.13(-0.19 to 0.45) | 4.54E-01 |  |  |
|  | 13 | Weighted mode | 0.13(-0.03 to 0.29) | 1.33E-01 |  |  |
| Body mass index | 10 | MR Egger | 0.37(-0.88 to 1.62) | 5.75E-01 | 0.006 | 0.456 |
|  | 10 | Weighted median | 0.96(0.60 to 1.32) | 1.59E-07 |  |  |
|  | 10 | IVW | 0.85(0.50 to 1.20) | 1.82E-06 | 0.006 |  |
|  | 10 | Simple mode | 1.24(0.55 to 1.93) | 6.35E-03 |  |  |
|  | 10 | Weighted mode | 1.12(0.52 to 1.73) | 5.26E-03 |  |  |

Beta (β), 95% confidence interval (CI), and P values were calculated for the respective method of MR analysis. The heterogeneity was tested by Cochran’s Q, including IVW and MR Egger methods. Horizontal pleiotropy was evaluated by MR Egger intercept. **Abbreviations:** nSNPs, number of single nucleotide polymorphisms; MR, Mendelian randomization; IVW, inverse-variance weighted.

**Supplementary Table S7** Mendelian Randomization and Sensitivity Analyses of Filtered Coffee on Mediators

| **Exposures** | **nSNPs** | **Methods** | **β (95% CI)** | **P value** | **P (heterogeneity)** | **P (pleiotropy)** |
| --- | --- | --- | --- | --- | --- | --- |
| Hypertension | 3 | MR Egger | 0.05(-3.14 to 3.24) | 9.79E-01 | 0.022 | 0.942 |
|  | 3 | Weighted median | 0.21(-0.01 to 0.44) | 6.56E-02 |  |  |
|  | 3 | IVW | 0.20(-0.07 to 0.47) | 1.53E-01 | 0.071 |  |
|  | 3 | Simple mode | 0.32(-0.01 to 0.64) | 1.94E-01 |  |  |
|  | 3 | Weighted mode | 0.27(-0.03 to 0.58) | 2.16E-01 |  |  |
| Triglycerides | 4 | MR Egger | -1.40(-2.33 to -0.47) | 9.89E-02 | 0.155 | 0.093 |
|  | 4 | Weighted median | -0.02(-0.17 to 0.13) | 8.11E-01 |  |  |
|  | 4 | IVW | 0.04(-0.26 to 0.33) | 8.11E-01 | <0.001 |  |
|  | 4 | Simple mode | 0.12(-0.28 to 0.53) | 5.94E-01 |  |  |
|  | 4 | Weighted mode | -0.11(-0.26 to 0.04) | 2.41E-01 |  |  |
| High-density lipoprotein cholesterol | 4 | MR Egger | 1.20(0.48 to 1.91) | 8.16E-02 | 0.364 | 0.068 |
|  | 4 | Weighted median | -0.05(-0.22 to 0.11) | 5.14E-01 |  |  |
|  | 4 | IVW | -0.11(-0.37 to 0.15) | 4.14E-01 | 0.002 |  |
|  | 4 | Simple mode | -0.38(-0.77 to 0.02) | 1.61E-01 |  |  |
|  | 4 | Weighted mode | 0.07(-0.08 to 0.21) | 4.28E-01 |  |  |
| Low-density lipoprotein cholesterol | 4 | MR Egger | 0.69(-0.08 to 1.45) | 2.22E-01 | 0.945 | 0.250 |
|  | 4 | Weighted median | 0.05(-0.10 to 0.20) | 5.18E-01 |  |  |
|  | 4 | IVW | 0.07(-0.06 to 0.19) | 2.92E-01 | 0.443 |  |
|  | 4 | Simple mode | -0.05(-0.30 to 0.21) | 7.50E-01 |  |  |
|  | 4 | Weighted mode | 0.16(-0.01 to 0.33) | 1.55E-01 |  |  |
| Body mass index | 3 | MR Egger | 0.39(-0.29 to 1.08) | 4.64E-01 | 0.783 | 0.588 |
|  | 3 | Weighted median | 0.13(0.01 to 0.25) | 3.31E-02 |  |  |
|  | 3 | IVW | 0.13(0.03 to 0.24) | 1.47E-02 | 0.724 |  |
|  | 3 | Simple mode | 0.09(-0.07 to 0.24) | 3.86E-01 |  |  |
|  | 3 | Weighted mode | 0.16(0.02 to 0.29) | 1.56E-01 |  |  |

Beta (β), 95% confidence interval (CI), and P values were calculated for the respective method of MR analysis. The heterogeneity was tested by Cochran’s Q, including IVW and MR Egger methods. Horizontal pleiotropy was evaluated by MR Egger intercept. **Abbreviations:** nSNPs, number of single nucleotide polymorphisms; MR, Mendelian randomization; IVW, inverse-variance weighted.

**Supplementary Table S8** Mendelian Randomization and Sensitivity Analyses of Instant Coffee on Mediators

| **Exposures** | **nSNPs** | **Methods** | **β (95% CI)** | **P value** | **P (heterogeneity)** | **P (pleiotropy)** |
| --- | --- | --- | --- | --- | --- | --- |
| Hypertension | 4 | MR Egger | -0.08(-1.26 to 1.09) | 9.01E-01 | 0.105 | 0.859 |
|  | 4 | Weighted median | 0.02(-0.08 to 0.13) | 6.79E-01 |  |  |
|  | 4 | IVW | 0.04(-0.07 to 0.14) | 5.17E-01 | 0.204 |  |
|  | 4 | Simple mode | 0.01(-0.16 to 0.18) | 9.22E-01 |  |  |
|  | 4 | Weighted mode | 0.01(-0.16 to 0.19) | 8.94E-01 |  |  |
| Triglycerides | 2 | IVW | 0.12(-0.23 to 0.47) | 4.87E-01 | <0.001 | NA |
| High-density lipoprotein cholesterol | 2 | IVW | -0.12(-0.41 to 0.17) | 4.07E-01 | <0.001 | NA |
| Low-density lipoprotein cholesterol | 2 | IVW | 0.08(-0.001 to 0.16) | 5.39E-02 | 0.796 | NA |
| Body mass index | 2 | IVW | 0.10(0.04 to 0.17) | 2.33E-03 | 0.433 | NA |

Beta (β), 95% confidence interval (CI), and P values were calculated for the respective method of MR analysis. The heterogeneity was tested by Cochran’s Q, including IVW and MR Egger methods. Horizontal pleiotropy was evaluated by MR Egger intercept. When only 2 instrumental variables existed, the pleiotropy test was not applicable, and the MR analysis along with heterogeneity test were performed only using IVW method. **Abbreviations:** nSNPs, number of single nucleotide polymorphisms; MR, Mendelian randomization; IVW, inverse-variance weighted.
